# Work-aged stroke survivors’ psychosocial challenges narrated during and after participating in a dialogue-based psychosocial intervention: a feasibility study

**DOI:** 10.1186/1472-6955-12-22

**Published:** 2013-09-25

**Authors:** Randi Martinsen, Marit Kirkevold, Berit Arnesveen Bronken, Kari Kvigne

**Affiliations:** 1 Faculty of Medicine, Institute of Health and Society, Department of Nursing Science, University of Oslo, Oslo, Norway; 2 Faculty of Public Health, Department of Nursing and Mental Health, Hedmark University College, Elverum, Norway

**Keywords:** Stroke, Work-aged stroke survivors, Marginalisation, Feasibility study, Complex intervention, Qualitative method, Hermeneutic-phenomenological, Rehabilitation

## Abstract

**Background:**

Studies point to the lack of psychosocial support and rehabilitation services that are adjusted to the work-aged stroke survivors’ specific needs in order to promote psychosocial well-being. The aim of the study was to illuminate the psychosocial challenges work-aged participants (i.e. aged 18–67 years) thematised during and after participating a dialogue-based psychosocial intervention during the first year following a stroke.

**Methods:**

The study was a feasibility study guided by the UK Medical Research Council Framework for developing and evaluating complex interventions. Qualitative data from in-depth interviews with fourteen stroke-survivors aged 33–66 years, researcher field notes and log notes written during the intervention were analysed applying a hermeneutic-phenomenological approach.

**Results:**

The stroke and its consequences had a substantial impact on family and work life. Their experiences were summarised in the two themes *The threat of becoming marginalised in family life* and *The threat of becoming marginalised in work life.*

**Conclusion:**

Life as a work-aged stroke survivor was experienced as challenging and created a threat of becoming marginalised in family and work life. The study highlights the need to understand the specific psychosocial challenges and needs facing work-aged stroke survivors’ in order to promote their psychosocial well-being. More research is needed concerning specific life-span challenges amongst work-aged stroke survivors in order to further develop appropriate interventions that helps address this issue.

## Background

The mean age of persons suffering from stroke in Norway is 76 years [1]. However, stroke may appear at any age [2]. It is estimated that approximately 25 percent of the stroke population is work-aged, i.e. below 65 years [3,4]. The seriousness and consequences of a stroke vary widely and may affect both physical functioning and psychosocial well-being [5]. Many stroke survivors experience emotional difficulties, such as anxiety and depression, both early on and later in the rehabilitation process [6-8]. Cognitive challenges, fatigue, social isolation and loss of self-confidence and control are frequent [5,9-11].

Although the challenges that older and younger stroke survivors experience overlap considerably, some studies suggest specific challenges to the work-aged stroke survivors, particularly with regard to relationships within and external to the family, marriage and parental roles and financial challenges [12-15]. Amongst work-aged stroke survivors, the psychosocial factors appear to have at least as great an impact on life after stroke as the physical consequences [13,16].

Work positively influences identity, self-esteem and psychosocial well-being. However, a limited number of stroke survivors return to work, indicating that residual stroke symptoms influence their ability to continue work as well as the ability to find new employment [17-21]. A successful return to work might have a positive impact on psychosocial well-being [19,22].

Studies point to the lack of psychosocial support, lack of information and rehabilitation services that are adjusted to the work-aged stroke survivors’ specific needs [5,13,23-25]. Because of the documented need for psychosocial support after stroke, our research group conducted a feasibility study to explore the usefulness of a dialogue-based psychosocial intervention. In this paper, we focus on the experiences of the work-aged participants who were included in the intervention. The aim was to illuminate the psychosocial challenges work-aged participants thematised during and after participating a dialogue-based psychosocial intervention during the first year following a stroke. Work-aged participants are defined as stroke survivors between the ages of 18 and 67 years.

## Methods

### Design

This study, conducted in Norway between May 2007 and October 2008, explored the usefulness of a complex nursing intervention. It was conducted according to the principles suggested by the UK Medical Research Council Framework [26]. As part of assessing the feasibility of the intervention, we examined the work aged participants’ experiences of psychosocial challenges and needs in order to refine the protocol before entering a full-scale randomised controlled trial (RCT).

The overall aim of the intervention was to promote psychosocial well-being. The intervention was planned to consist of eight one to two hour dialogue-based encounters over a six months period. The encounters focused on the psychosocial consequences of the stroke. Each meeting had a preplanned guiding topical outline, based on existing knowledge of psychosocial needs during the rehabilitation trajectory. The participants were invited to tell about the issues that were most important to them at the time and the guiding topical outline was adjusted based on the participants’ individual needs. Family members were invited to take part in one or more of the intervention encounters, subject to approval by the participants. The intervention is described in detail elsewhere [27].

### Recruitment

Norwegian speaking stroke survivors aged above 18 years were recruited to participate in the intervention by a designated nurse during hospitalisation or by the home care services. They were invited to participate in the intervention if they had suffered a stroke within the last eight weeks, were medically stable, were judged to have adequate cognitive functioning and to benefit from the intervention by their physician. A speech-and-language therapist assisted in recruitment of persons with moderate to severe aphasia. Oral and written information was adjusted linguistically, and consent was obtained. A speech-and-language therapist assessed language abilities according to production and understanding of oral and written language, reading, and writing prior to inclusion.

### Data collection

Background information, consisting of demographic data and medical information, were collected by the recruitment personnel.

During the intervention, the health workers who carried out the intervention wrote log notes after each encounter noting the themes addressed in the encounters, specific reactions during the encounters (if any), whether other participants (i.e. next of kin) were present and the health workers’ own reflections.

Two to four weeks after conclusion of the intervention, three members of the research team interviewed the participants in-depth. All members of the research team were nurses; one was a senior lecturer and two were doctoral students. All had previous experience conducting qualitative interviews. Two had conducted qualitative interviews with stroke survivors previously, one was specially trained to conduct interviews with stroke survivors’ suffering from aphasia. The principal investigator was a professor who supervised the research team during the interview processes. A speech-and-language therapist supervised the researcher interviewing the participants with aphasia.

The interviews had a guiding thematic outline, which focused on the experiences of the participants, including their present lives, their thoughts and reflections concerning their lives and psychosocial needs following the stroke. The interviews had open-ended questions as a point of departure, like “Please tell about your life at the present”. Probing was used to encourage the participants to expand their descriptions related to their psychosocial well-being after participating in the intervention, for instance: “Can you tell me whether participating in the intervention has made a difference or not in relation to your well-being?” The interviews with the participants with aphasia were carefully prepared and carried out by a specially trained nurse, using the approach Supported conversations for adults with aphasia [28].

The interviews were audiotaped and the researchers wrote reflection notes after the interviews. In addition, the interviews with the participants suffering from aphasia were video-recorded to better capture both their verbal and non-verbal expressions.

Five participants were interviewed individually in a city-based Learning and Mastery Centre, two in a conference room at a hospital, five in their homes and two in a nursing home, according to their preferences. No family members took part in the in-depth interviews.

### Participants

Fourteen of the twenty-five stroke survivors who participated the feasibility study, met the inclusion criteria of being work-aged (i.e. aged 18–67 years). Eleven men and three women, aged 33 to 66 years (mean age 54.6) were included in this subset of the study. The time range between inclusion in the study and the first intervention encounter differed for practical reasons. Consequently, the time between the stroke incident and the in-depth interviews ranged from seven to ten months. For participants suffering from aphasia the intervention period was prolonged until twelve months post stroke, as they were unable to complete the intervention within the original timeframe due to their communication challenges. In these cases, the time from stroke onset to the in-depth interviews ranged from eleven to thirteen months.

Six of the participants were diagnosed with thrombosis in the right hemisphere. Four of these had paresis of the left side, one had reduced strength in the left side, and one had no visible physical symptoms. Four of the participants were initially diagnosed with a thrombosis in the left hemisphere of whom two suffered paresis on the right side. One had a slight numbness and reduced strength in the right side, while the last one had no visible physical symptoms. Three of the participants were diagnosed with a haemorrhage in the left hemisphere. One of these had paresis on the right side, one had reduced strength on the right side, while the last one had no visible physical symptoms. One participant was diagnosed with a thrombosis and haemorrhage in the left hemisphere and had paresis on the right side.

Four of the participants suffered from visual deficits. Three of the participants had neglect, while two had concentration problems and/or laps of memory. Two of the participants were initially diagnosed with mild aphasia, while six were diagnosed with moderate to severe aphasia.

The participants lived in rural and urban areas of southern Norway. Seven of the participants were married, two were cohabitants, three were divorced, and two were single. Five of the participants had minor children living at home. All of the participants lived at home prior to the stroke, and all but two lived at home at the conclusion of the intervention. Eleven of the participants were employed part-time or full-time prior to the stroke onset, whereas the other three were unemployed or received social security benefits. Five of the participants had returned to part-time work at the conclusion of the study.

### Data analysis

The analysis was conducted applying a hermeneutic-phenomenological approach and consisted of three steps [29-31].

During the first analytical step, the recorded and transcribed interviews were read carefully as a whole to get an initial and holistic impression of the participants’ experiences. This step resulted in a narrative summary of the experiences of each participant. Next, the researchers’ notes and the health workers’ log notes were explored and new insights about the participants’ experiences added to the fourteen narratives. The compiled text for each case was then read carefully as a whole to get an initial and holistic impression of the participants’ experiences of psychosocial challenges and needs. This open, inductive reading suggested a naïve understanding of work-aged stroke survivors describing their lives as difficult and changed with respect to carrying out roles and responsibilities related to their family and work. We interpreted their descriptions as indications of possibly being in a marginalised situation defined as being placed on the periphery of society [32-34], in this case in the family and with regard to work. Marginalisation is related to personal identity and impacted by personal experiences and environmental factors [32]. Using this working definition, the second analytical step consisted of an inductive structural analysis of the narratives, guided by the following overall questions: What are the specific experiences and psychosocial needs of work-aged stroke survivors? Is the initial interpretive working hypothesis of possible marginalisation confirmed or disconfirmed upon further analysis?

During the structural analysis (the second analytic step) the narratives were divided into meaning units, condensed into detailed subthemes related to family and work in order to further explore the meaning of the text and to validate or invalidate the initial impression. Finally, during the third analytic step, we summarised and reflected on the inductively derived subthemes in order to develop a comprehensive understanding of the experience of being a work-aged stroke survivor. This resulted in a reformulation of the initial impression; we found that being a work-aged stroke survivor implies a threat of becoming marginalised in relation to family life and work life. During this step, the narratives were reread as a whole and reflected on in light of theory of marginalisation and the broader research literature on psychosocial aspects of stroke.

Each step of the analysis was discussed within the research group and consensus obtained before moving to the next step. An example of the analysis is shown in Table 1.

**Table 1:** Example of structural analysis process

**Meaning unit**	**Condensation**	**Sub-theme**	**Theme**
Increased need for rest and sleep, lack of memory complicate caring for own family. His need is to resume activity, to suffice in caring for those he lives together with.	A challenge to care for the family	*Unable to perform the caring role as before*	The threat of becoming marginalised in family life
	Difficult to care for the family		
Friends were making contact, but he was too exhausted for company. He missed the social life, but felt he had to manage organising his own life first.	To participate in social gatherings is exhausting	*Falling out of extended family activities*	
Difficult to adapt to a life without working and that others told her that she not was able to return to work.	Leave work involuntary	*Being forced to leave work*	The threat of becoming marginalised in work life
A meeting at the workplace changes her expectations. The employer tells her that she is fired and that she cannot come back to work.	Fired		
Hopes to be full-time worker within the next six months. He is unsure of how he could manage return to ordinary work. The leadership position demands a lot of travelling.	Challenging to return to ordinary work	*Struggling to meet work expectations*	

### Ethics

Ethical approval was obtained from the Regional Medical Ethics Committee and the Social Science Data Service in Norway. All participants provided written, informed consent prior to being enrolled in the intervention. A verbal consent was repeated during the intervention period [35]. All participants were assured of their confidentiality and the possibility to withdraw from the study at any time without consequences, as stated in the Helsinki Declaration [36].

## Results

Stroke was experienced as having a substantial impact on family and work life. *The threat of becoming marginalised in family life* and *The threat of becoming marginalised in work life s*ummarised the work-aged stroke survivors’ experiences during the first six months following a stroke, and is further described below.

### The threat of becoming marginalised in family life

The participants described challenges in terms of meeting expectations in family life following the stroke related to both the immediate and extended family, and friends. This theme is divided into the following two subthemes: *Unable to perform the caring role as before*, and *Falling out of extended family activities.*

#### Unable to perform the caring role as before

The struggle to participate in family life in the same manner as before the stroke raised numerous concerns. The reduced ability to collaborate with their partner in caring for their children made them feel like they were not part of the family. Although some expressed themselves as “restored” in a physical sense, they reported a need for greater emotional and social involvement in family life than what they currently felt were the case. Cognitive difficulties, emotional distress and lack of stamina affected their level of participation in daily home activities and prevented them from meeting their own expectations and/or those of the immediate family.

One of the male participants who lived in a nursing home, but had small children at home, expressed powerlessness as a father. The parental situation caused significant worries that dominated the first weeks of his hospital stay and continued throughout the follow-up period. The consequences of a severe stroke left him unable to care for himself and his partner was reluctant to take him home because of his extensive care needs. Although he expressed an understanding of her resistance to have yet another person to care for, he struggled with being on the periphery of his family, prevented from participating in the care for his children and supporting his partner in her caring role. The inability to fulfil his responsibility in family life led to the need for professional help to care for the children during some of the weekends in order for his partner to get some relief. This caused “terrible feelings” that worsened his situation. He realised that he had to abandon or renegotiate his previous family role. As his ability to manage and influence family life was limited; his role as a father was constricted, and he lacked the ability and opportunity to fulfil his expectations and desires:

*I will not be able to participate the children’s life like before. Time is gone when I was able to play together with my own children. I used to be strong like a horse. Could be together with my children for hours, and then go to work without having a rest*.

Some participants expressed that their changed mood, their inability to explain their situation and the children’s and/or spouse’s lack of patience could be a challenging ordeal that, for some, led to family conflicts. Some families did not communicate about how their life situation was affected when a family member had suffered from a stroke. This led to broken relationships or conflicts. One father talked a lot about an on-going conflict with his son during the intervention, which caused him a lot of worries. The father’s aphasia complicated their communication, resulting in misunderstandings and frustrations:

*I have problems with […] my son. Serious problems. He has not taken part in anything. Nothing has he taken part in! [Upset]. He has been angry […] It is like an explosion*.

Most of the participants highlighted the importance of information and support in order to understand the consequences of stroke and they emphasized that this was a means of helping both the participant and the family move on with life in the best possible way. The family’s lack of information concerning the impact of stroke contributed to the participants’ difficulties with caring for their children:

*They are looking at my shuffling walk, [and it is] not always easy to get understanding for all situations. I can’t keep up, can’t come along to everything or join everything. It’s not a problem to me, but it’s not always easy to get understanding for [the fact] that you need to rest and are not able to keep up all the time. They need information*.

The participants spent much time considering their inability to talk with their children about how symptoms influenced everyday life; their inability to support their children’s activities; or worries about their children’s inability to concentrate on their schoolwork. One father expressed his despair of the lack of concern by the children’s teachers and the school nurse:

Nobody has asked my children. The teachers haven’t asked my children. They know about my stroke. […] It’s a pity. It’s incredible […] She [the daughter] was [so scared] sent me sms every second hour [just saying] Hi! [to check if I was ok].

The inability to keep up the traditional parent role was also underscored by the fact that for two of the stroke survivors, their sons had to support them in carrying out basic mathematics. This demonstrated that not only were their roles constricted, but their roles were reversed. Contrary to their expectation of the opposite, the fathers experienced having to be cared for by their children. The fathers wanted to support their children, but were unable to manage because of cognitive difficulties. They found the situation difficult to explain to their children:

*I had anxiety talking with my kids about what had happened. I need energy to be capable to talk with them. I had enough with myself, so I didn’t manage, or I didn’t dare. I had anxiety*.

Some participants expressed how their lack of understanding influenced their motivation to meet the challenges and expressed the need to have someone to talk to in order to overcome the frustration of not being sufficiently motivated:

*I had a lot of questions about my own life. Why am I so tired? [ …] What is going on in my brain? [… ] So, I could understand myself in terms of […] [having suffered a stroke] and in a way get the answer of why I was changing […] emotionally*.

#### Falling out of extended family activities

Living in a family implies social gatherings beyond the immediate family. The extended family may include other relatives and/or close friends. The participants stated that it was challenging to participate in gatherings in the same manner that they did before the stroke. One of the participants was told by his wife:

*[…] that the in-laws wondered if I was outside or if I participated. I was sitting quietly and calmly in a corner, far away from the centre of activities*.

During a birthday party, the screaming children and the laughing families made him even more exhausted and tired. He participated in the gathering as a passive spectator, marginalising himself by withdrawing from the centre of activities. He preferred being at the periphery to manage the situation.

Cognitive challenges, including a lack of stamina, concentration problems and tiredness or exhaustion affected ordinary leisure activities and made it difficult for the participants to pursue interests and attend leisure gatherings. The situation resulted in activity avoidance to prevent unbearable situations of being exhausted afterwards. However, the fear of losing friends because of not participating was challenging:

*[…] Now I’m mostly together with my family. Before I was probably mostly together with friends. My circle of friends has decreased. In the beginning I didn’t want to [withdraw], I wanted to try to keep on just like before, but it [participating in social life] doesn’t work*.

The inability to drive a car also contributed to the experience of social isolation. Several participants struggled with whether they would be able to drive again, realising that the inability to drive kept them from participating in life as they had before the stroke onset. However, getting the driver’s licence back did not necessarily solve the problem. Despite obtaining a renewed driver’s license, a woman explained:

*Somehow, when I got it [driver’s license] back […] I was afraid of driving*.

The inability to drive caused stroke survivors to depend on others which further complicated their ability to participate and contribute socially on equal terms. Remaining at home or in a nursing home, involuntarily or as a voluntary safety measure, was like an environmental barrier that strained life and isolated most of these stroke survivors from an ordinary social life. Their new life evolved, in many ways, at the margins of the active and busy lives of their family and friends.

### The threat of becoming marginalised in work life

The theme *The threat of becoming marginalised in work life* reveals how the stroke influenced work life and how the participants experienced this as employees. These experiences are captured by the following two subthemes: *Being forced to leave work* and *Struggling to meet work expectations*.

#### Being forced to leave work

Although some of the participants expected to return to work, some were forced to leave work because of the consequences of stroke. A newly graduated woman had her stroke when she was newly employed. In a planned meeting at the conclusion of the rehabilitation process, this woman, who suffered aphasia, was called to meet her employer. In her mind, the purpose of the meeting was to plan her return to work. Instead, she was dismissed from the job, which stunned and upset her, because continuing in her newly acquired job had been a major goal for her rehabilitation. She received no warning and was not prepared for being fired. Struggling to express her chock and despair, due to her language problems, the participant expressed how this event disrupted her self-confidence and identity. Throughout the intervention period, she returned to this situation and struggled to receive help in order to fight her employer and get her job back.

A female participant close to retirement age wished to return to work, but experienced that health care professionals decided that she should retire. She found the decision difficult to accept and experienced that an important part of her adult life and her day-to-day experiences had completely changed:

*I liked my colleagues and everything that happened. [Now, after retirement] you, you, just get up and only [do nothing] […]*.

Her social life and work life had been interrupted, and she felt marginalised and disempowered.

Participants with children at home, who in this study were men only, were preoccupied with their roles as breadwinners. The financial responsibility highlighted the importance of returning to work. The informants spoke about *when* they would go back to work, their hopes of managing work life after the stroke, the struggle to imagine what would happen if they were unable to return to work and the threat of becoming impoverished in the future.

#### Struggling to meet work expectations

As the previous theme underscored, the consequences of stroke complicated returning to work. However, two of the participants had employers who arranged the work environment to accommodate continued employment by protecting them from noise, allowing for flexibility and creating rest periods during the day. One of the participant’s boss had suffered from a stroke himself and understood the need for facilitating a post-stroke-friendly work place. The health worker’s log note illuminated the situation:

*He […] works two days a week now. […] Is very happy about that. […] He has to ask his doctor for a new sick leave. Hopes he can work 60% from next month. It is okay for the employer. It is going well to communicate with colleagues. Feels included and taken care of*.

One of the male participants, who had been on sick leave for a long time before the stroke and had not worked full-time for three years, was planning to return to work and hoped to be a full-time worker within the next six months. However, he was unsure of how he could cope with the working situation. He said it would be challenging to return to ordinary work as a manager because of the difficulty of working without becoming exhausted. Additionally, the leadership position demanded his travelling to visit customers, which he felt would be very difficult for him. He realised that his position had to change. His cognitive and physical impairment affected his self-esteem, which left him in a marginalised position that made it difficult for him to meet his work life expectations.

Another participant who returned to half-time work during the intervention struggled with whether he could work full-time without becoming exhausted. He described some uncomfortable experiences when his employer questioned his ability to return to work:

*They are asking me at work: When do you think you will start to work a hundred percent again? [ …] I can’t say anything about that. I can’t be specific*.

Further on, one of the stroke survivors experienced a situation in which his colleague, who was temporarily acting in his position as day-to-day head of the company, was collecting information concerning his workday performance in order to have him dismissed:

I can’t think of it. He [the employer] wouldn’t give me new clients. I don’t understand that he claims that I don’t manage. I’m going to convince him that I manage!

The employer’s willingness to maintain income or avoid a salary reduction which was the case to two of the stroke survivors released them from economic troubles. Being marginalised in terms of performing usual work roles left some participants in a challenging financial position, and caused emotional distress. They contemplated whether it would ever be possible to resume regular work. The goal was to continue being the primary wage earner to avoid impoverishing the family.

## Discussion

Overall, this study suggests that work-aged stroke survivors experience substantial threats of becoming marginalised, both in family and work life. To the best of our knowledge, work-aged stroke survivors’ experiences of threats of marginalisation have been poorly explored and discussed in the literature.

Marginalisation can lead to an experience of becoming “invisible” compared to those who find themselves in the centre of events, which in turn causes negative consequences that threaten health and psychosocial well-being [32]. Throughout life, multiple sources of marginalisation may occur, influencing one’s personal sense of worth [32,37]. The social course of the marginalisation process can lead to role constriction and inability to fulfil various social functions, delegitimation and a lack of validation as a person, and being relegated to the position of being “sick”, impoverished and socially isolated [38]. This study confirms that role constrictions can negatively affect stroke survivors. Suffering from invisible symptoms, as many of the study participants did, might increase delegitimation and create a threat of marginalisation as family and friends have difficulties understanding or recognizing these symptoms and the problems and consequences they create [13,39].

All participants experienced a reduced ability to maintain the roles of parent, partner and breadwinner. These findings differ from and extend those of Röding et al. [13] who found that challenges related to caring for children and housework were specific to female stroke survivors. None of the women in this study had children living at home; thus, our data cannot confirm gender differences with respect to the caring role. However, our findings suggest that the men did experience marginalisation in their roles as fathers of small children and teenagers. This outcome might be a result of recent changes in caring roles in Norway in which mothers and fathers have more equal roles in caring for children. Similar trends are seen elsewhere [40]. The men also expressed economic concerns and worried about their ability to take care of their family economically. This is consistent with the findings of Röding et al. [13].

Our findings suggest that challenges related to work life may be experienced as a threat to identity affecting psychosocial well-being. This is the case even for those who are near retirement. The two women in this study, who had full-time work prior to their stroke, were told by others that they could not continue to work. According to Ahlgren and Hammarström [41], women are more likely to have decisions made for them during rehabilitation than men. Involving the stroke survivor, irrespective of gender and age, in decisions concerning leaving work appears to be essential because both women and men strongly identify life with work, and consequently experience leaving work as disrupting [18,40]. Physical dysfunction should not affect an individual’s opportunity to make decisions [11]. Being uninvolved could contribute negatively to psychosocial well-being by making life after a stroke more challenging and by threatening one’s right to self-determination and empowerment.

In this study, marginalisation was not an explicit theme introduced by the intervention. However, the flexible outline of the intervention permitted discussing the participants’ own needs and challenges, which resulted in dialogues about a number of issues related to marginalisation in their lives. This study revealed the importance of rehabilitation professionals being aware of the threat of marginalisation that arises when work-aged stroke survivors struggle to meet family and work life responsibilities and expectations. Such awareness may likely increase the ability of health workers to help work-aged survivors manage the psychosocial challenges after a stroke. Knowing that the consequences of stroke can be long lasting [42] and lead to marginalisation, our study suggests that an individualised, dialogue-based intervention that invites stroke survivors to talk about their family and work life could be a positive contribution to enhancing psychosocial well-being [43-45]. In contrast, the lack of opportunities to talk about experiences and challenges following a stroke may threaten psychosocial well-being. However, further research is needed to evaluate the effect of such an intervention.

The threat of becoming marginalised identified in this study does not signify that all work-aged people living with stroke will be marginalised. However, it is important that health care professionals are aware of the threat of becoming marginalised. Although it might not necessarily be helpful to stroke survivors to be introduced for the concept of marginalisation, especially early in the rehabilitation process, using the concept as a professional, sensitising tool, which points to challenges that often escape the attention of health care professionals, might be useful. The most pertinent approach, according to our findings, is to encourage and support the stroke survivors’ innate ability to narrate their experiences and new situation.

This study, which was part of a feasibility and piloting stage to prepare for a randomised study, is consistent with the development of complex interventions suggested by the UK Medical Research Council [26]. This framework recommends the feasibility to be assessed before proceeding to a full trial. A feasibility evaluation will maximise the possibility of identifying effective components and securing the necessary development of the intervention before launching a RCT. The issues uncovered in this study, will assist us in the further development of the intervention in order to address the needs of work-aged survivors.

### Limitations

Several standardised instruments, measuring quality of life, depression, anxiety and psychosocial well-being, were tested in the feasibility study for their appropriateness in a RCT. However, because not all participants were able to complete them, they are not included in this qualitative evaluation of the intervention study. We did not include standardised instruments for stroke severity, physical and cognitive functioning and personality, but relied on the responsible clinicians’ descriptions of the participants at the time of inclusion. This is a limitation in this study and will be changed in the follow-up RCT.

The health workers’ log notes differed with respect to the descriptions of the themes addressed in the encounters, specific reactions during the encounters and the health workers’ own reflections. However, the triangulation of different data collection methods that were utilised assured us that we had rich descriptions of the participants’ experiences.

The sample size of fourteen participants is limited and our participants do not necessarily represent the general population of work-aged stroke survivors. Nevertheless we believe that our findings are relevant for many work-aged stroke survivors, as they are consistent with and extend other qualitative studies.

Validation was promoted by having several researchers performing the analysis and by maintaining open and continuous discussions within the research group in order to reach consensus about the findings.

## Conclusion

Life as a work-aged stroke survivor was experienced as challenging and created a risk of becoming marginalised in family and work life. The study highlights the need to understand the experience of becoming marginalised in order to promote work-aged stroke survivors’ psychosocial well-being. More research is needed concerning specific life-span challenges amongst work-aged stroke survivors in order to further develop appropriate interventions that address this issue.

## Competing interests

The authors declare that they have no competing interests.

## Authors’ contributions

RM, MK, BAB and KK were responsible for the study conception and design, and data analysis. RM and MK were responsible for the manuscript preparation. All authors contributed to the critical revisions of the manuscript, read and approved the final manuscript.

## Authors’ information

RM: RN, M.N.S., Doctoral student,

Faculty of Medicine, Institute of Health and Society, Department of Nursing Science, University of Oslo, and Faculty of Public Health, Department of Nursing and Mental Health, Hedmark University College

MK: RN, Ed.D., Professor,

Faculty of Medicine, Institute of Health and Society, Department of Nursing Science, University of Oslo

BAB: RN, M.N.S., Doctoral student,

Faculty of Medicine, Institute of Health and Society, Department of Nursing Science, University of Oslo, and Faculty of Public Health, Department of Nursing and Mental Health, Hedmark University College

KK: RN, PhD., Associate Professor,

Faculty of Public Health, Department of Nursing and Mental Health, Hedmark University College.

## Pre-publication history

The pre-publication history for this paper can be accessed here:

http://www.biomedcentral.com/1472-6955/12/22/prepub
